# A Connectomic Analysis of the Human Basal Ganglia Network

**DOI:** 10.3389/fnana.2017.00085

**Published:** 2017-09-26

**Authors:** Alberto Cacciola, Alessandro Calamuneri, Demetrio Milardi, Enricomaria Mormina, Gaetana Chillemi, Silvia Marino, Antonino Naro, Giuseppina Rizzo, Giuseppe Anastasi, Angelo Quartarone

**Affiliations:** ^1^IRCCS Centro Neurolesi “Bonino Pulejo”, Messina, Italy; ^2^Department of Biomedical, Dental Sciences and Morphological and Functional Images, University of Messina, Messina, Italy

**Keywords:** connectivity, connectome, constrained spherical deconvolution, diffusion magnetic resonance imaging, neural pathways, white matter, tractography

## Abstract

The current model of basal ganglia circuits has been introduced almost two decades ago and has settled the basis for our understanding of basal ganglia physiology and movement disorders. Although many questions are yet to be answered, several efforts have been recently made to shed new light on basal ganglia function. The traditional concept of “direct” and “indirect” pathways, obtained from axonal tracing studies in non-human primates and post-mortem fiber dissection in the human brain, still retains a remarkable appeal but is somehow obsolete. Therefore, a better comprehension of human structural basal ganglia connectivity *in vivo*, in humans, is of uttermost importance given the involvement of these deep brain structures in many motor and non-motor functions as well as in the pathophysiology of several movement disorders. By using diffusion magnetic resonance imaging and tractography, we have recently challenged the traditional model of basal ganglia network by showing the possible existence, in the human brain, of cortico-pallidal, cortico-nigral projections, which could be mono- or polysynaptic, and an extensive subcortical network connecting the cerebellum and basal ganglia. Herein, we aimed at reconstructing the basal ganglia connectome providing a quantitative connectivity analysis of the reconstructed pathways. The present findings reinforce the idea of an intricate, not yet unraveled, network involving the cerebral cortex, basal ganglia, and cerebellum. Our findings may pave the way for a more comprehensive and holistic pathophysiological model of basal ganglia circuits.

## Introduction

The current view of basal ganglia neurophysiology is largely based on data from rodents and non-human primates using various tract-tracing methods for the study of monosynaptic connections combined with immunohistochemistry and *in situ* hybridization (Albin et al., 1989; DeLong, 1990). According to this view, the cerebral cortex is widely connected with the basal ganglia via two major projection systems; the so-called “direct” and “indirect” striatofugal pathways originating from segregated populations of striatal projection neurons that exert opposite effects upon basal ganglia outflow. In this model, cortical efferents terminate on specific subcortical areas, which in turn project to the same cortical area that started the initial impulse. The main evidence of this morpho-functional segregation was originally provided for the dorsal striatum, which was classically considered as the main input station in the basal ganglia circuitry (Albin et al., 1989).

In addition to the striatum, a glutamatergic hyper-direct pathway connecting the cerebral cortex and subthalamic nucleus (STN) has been described in the monkey (Nambu et al., 2002) and would be responsible for a rapid signal transmission from the cortex to the internal segment of the globus pallidus (GPi) (Nambu et al., 2000).

Classically, the anatomical description of the basal ganglia circuitry has been based on axonal tracing in non-human primates and post-mortem fiber dissection in the human brain. However, in the last 20 years, Diffusion Tensor Imaging (DTI) tractography has been used as an anatomical “virtual dissector” for tracking *in vivo* and non-invasively neural connectivity in the human brain (Basser et al., 1994; Henderson, 2012; Nunnari et al., 2014), by measuring anisotropy diffusion of water molecules (Mori and Van Zijl, 2002).

A DTI study in humans demonstrated that projections from pre-supplementary motor area (pre-SMA) reach only the anterior and middle part of the caudate nucleus and putamen, while the SMA and primary motor area (M1) send dense efferents to the posterior part of putamen and a few to its middle part (Lehéricy et al., 2004a). Further investigations demonstrated that, in humans, the caudate head is connected with several frontal areas (Lehéricy et al., 2004b; Draganski et al., 2008). On the other hand, the caudate tail is technically difficult to study in humans because of its poor detectability with neuroimaging techniques. Studies on primates suggest a major connectivity with temporal areas (Yeterian and Van Hoesen, 1978). This topographical distribution of inputs appears to be maintained in striatal efferents in macaques (François et al., 1994) and in pallidal and nigral efferents in monkeys (*Cebus Apella*) (Hoover and Strick, 1999).

The existence of the hyper-direct pathway in humans was first demonstrated in a study assessing the STN connectivity with the motor, associative, and limbic brain areas, based on structural and functional connectivity analysis (Brunenberg et al., 2012). The STN is connected with the primary motor cortex, premotor and supplementary motor cortex, and the somatosensory cortex, in lines with studies in non-human primates (Nambu et al., 2002; Brunenberg et al., 2012; Milardi et al., 2015).

In a recent study, we have challenged the traditional model of the basal ganglia network by showing evidence of cortico-pallidal projections in humans by means of Constrained Spherical Deconvolution (CSD) based tractography, which allows to successfully resolve multiple fiber populations insisting over the same voxel and to overcome most of the DTI model issues.

Indeed, we identified a direct cortico-pallidal pathway connecting both the GPi and the external segment of the globus pallidus (GPe) with several cortical regions and running through the internal capsule (Milardi et al., 2015). This pathway may represent an additional route for upstream cortical regulation on basal ganglia circuitry, bypassing the striatum and paralleling the hyperdirect pathway (Smith and Wichmann, 2015).

Furthermore, we showed tractographic evidence of a complementary direct cortico-substantia nigra (SN) pathway paralleling the direct, indirect, hyperdirect, and cortico-pallidal systems (Cacciola et al., 2016a). Several studies revealed cortico-nigral connections in rodents (Sesack et al., 1989), primates (Frankle et al., 2006) and humans (Kwon and Jang, 2014; Cacciola et al., 2016a). Although in animals it was shown that this direct pathway may represent a glutamatergic cortical regulator of SN activity (Kornhuber et al., 1984), in humans its function and physiological significance are still unknown.

It is worthy to note that diffusion-based tractography is not sufficient to provide anatomical evidence of the existence of a specific pathway, if used alone (Jbabdi and Johansen-Berg, 2011). Indeed, it calculates the highest mathematical probability that water diffuses in a given direction, thus inferring the preferential water diffusivity directionality along white matter bundles. In addition, it should be considered that tractographic reconstruction is not able to clearly distinguish mono- from polysynaptic connections as well as the directionality (afferent or efferent) of the signal transmission (Chung et al., 2011; Parker et al., 2013). Nevertheless, tractography is the only method available to study non-invasively anatomical connectivity in the human brain *in vivo*.

In the present study, we used a robust diffusion signal model, namely the CSD, for tracking and assessing the connectivity patterns and topographical organization of the basal ganglia network.

## Materials and methods

### Participants

We recruited 15 human subjects (9 women, mean age 29 years; age range 25–32 years) with no previous history of disease. The study followed the tenets of the Declaration of Helsinki; written informed consent was signed from all included subjects after explanation of the nature and possible consequences of the procedure. The study was approved by the institutional review board of IRCCS Bonino Pulejo, Messina, Italy (Scientific Institute for Research, Hospitalization and Health Care).

### Data acquisition

The study was performed with a 3T Achieva Philips scanner equipped with a 32-channels SENSE head coil. In each subject, a structural 3D high-resolution T1 weighted Fast Field Echo (FFE) sequence was acquired using the following parameters: repetition time 25 ms; echo time 4.6 ms; flip angle 30°; FOV 240 × 240 mm^2^; isotropic voxel size 1 mm. The acquisition time was 6 min. Furthermore, a 3-D high resolution T2 weighted Turbo Spin Echo (TSE) sequence was obtained using the following parameters: repetition time 2,500 ms; echo time 380 ms; FOV 250 × 250 mm^2^; in plane reconstruction matrix 312 × 312; slice thickness 0.8 mm. The acquisition time was 9 min and 38 s.

The use of 3D TSE sequence allowed to obtain high-resolution images with a relative short acquisition time, as well as to provide a fine representation of the iron loaded nuclei due to T2^*^ effect linked with the use of a very long echo-time.

Furthermore, a Diffusion weighted dataset (DWI) was obtained with a single-shot EPI sequence using the following parameters: repetition time 11,884 ms, echo-time 54 ms, FOV 240 × 240 mm^2^, isotropic voxel size of 2 mm. Thirty-two diffusion encoded volumes were acquired using a b-value of 1,000 s/mm^2^; in addition, two un-weighted b0 volumes were acquired, one of which with inverted phase-encoding direction for post-acquisition distortion corrections.

### DWI pre-processing and co-registration

All diffusion images were corrected for motion as well as for Eddy Currents distortion artifacts using a combination of topup and eddy FMRIB Software Library (FSL) tools (http://fsl.fmrib.ox.ac.uk/fsl/fslwiki/); rotational part of transformations were later applied to individual gradient directions. High Resolution T1 and T2 images were then co-registered to preprocessed DWIs using a pipeline outlined in Besson et al. (2014): for each subject, cerebrospinal fluid (CSF) probability maps were estimated from b0 image as well as from T1w volume using New Segment SPM8 utility (http://www.fil.ion.ucl.ac.uk/spm/software/spm8/). Then, CSF of structural scan was warped to match CSF estimated from b0 image using FLIRT (FMRIB's Linear Image Registration Tool) and FNIRT (FMRIB's non-linear registration tool). Subsequently, estimated deformation field was applied to T1w volume. In a separate stage, an affine transformation, using FLIRT FSL command, was performed in order to co-register the T2w volume to T1w scan; eventually, the same warping field previously obtained was applied to this map. In this way, we obtained an overlap between structural images and DWIs as accurate as possible.

### Nuclei segmentation and cortical parcellation

SN and STN segmentation was manually performed on T1w and T2w co-registered volumes by a skilled neuroradiologist, as outlined in a previous our work (Milardi et al., 2016a).

Cortical and subcortical reconstruction and volumetric segmentation were performed on co-registered T1 images with the Freesurfer image analysis suite, which is documented and freely available for download online (http://surfer.nmr.mgh.harvard.edu/). Briefly, this processing involves motion correction and averaging (Reuter et al., 2010) of T1 weighted images, removal of non-brain tissue using a hybrid watershed/surface deformation procedure (Ségonne et al., 2004), segmentation of the subcortical white matter and deep gray matter volumetric structures (Fischl et al., 2004), tessellation of the gray matter white matter boundary, automated topology correction (Ségonne et al., 2007), and surface deformation following intensity gradients to optimally place the gray/white and gray/CSF borders at the location where the greatest shift in intensity defines the transition to the other tissue class (Fischl and Dale, 2000). Once the cortical models were completed, parcellation of the cerebral cortex into units with respect to gyral and sulcal structure (Desikan et al., 2006) was performed. Subsequently, the obtained parcellations and segmentations of each subject were visually inspected and, if needed, manually edited.

### Tractography

To model diffusion signal, we used a modified High Angular Resolution Diffusion Imaging (HARDI) technique called non-negative CSD: this technique estimates the fiber Orientation Distribution Function (fODF) directly from deconvolution of DW signal with a reference single fiber response function (Tournier et al., 2007, 2008). fODF estimation and tractography were performed using MRtrix software (http://www.mrtrix.org).

By using CSD to extract local fiber orientations we could overcome partial volume effects associated with DTI. Higher b-values permit to resolve smaller angles among fibers (Alexander and Barker, 2005; Tournier et al., 2007); on the other hand, they require longer acquisition times thus increasing the probability to find motion and eddy currents related artifacts. Thus, we preferred a lower b-value in order to obtain a reasonable quality speed trade-off.

In our study, spherical harmonic degree was fixed equal to six in order to obtain robustness to noise. During tractographic reconstruction, tracking was stopped in one of the following conditions: step size = 0.2 mm, maximum angle = 10°, minimal fODF amplitude = 0.15. The latter parameter allowed to obtain more accurate reconstructions avoiding streamlines to enter GM in deep or passing through CSF; indeed, in those regions, estimated fODF amplitudes are lower than such cut-off. This is a more conservative choice with respect to usual standards, since we preferred to underestimate fiber bundles in order to have more consistent reconstructions (Descoteaux et al., 2009; Tournier et al., 2011; Milardi et al., 2017).

Whole brain probabilistic tractography was run by generating 10 million tracts using the above-mentioned criteria for streamline creation and end. At this stage, a moderately dilated version of WM mask was used both as seed and termination mask, to further reduce the risk of implausible streamlines to be generated.

### Connectogram construction

To create connectograms of right and left subcortical nuclei (caudate, putamen, GP, STN, and SN), the following pipeline was adopted: for each nucleus, MRtrix tckedit command (*include* option) was employed to filter the tracks of interest. At the same time, each structure obtained from Freesurfer segmentation was extracted and included within the same command (*include* option) to isolate the specific tracks linking the basal ganglia to the related area. The same process was repeated for all ROIs obtained from Freesurfer. Subsequently, in-house scripts built in Matlab (http://www.mathworks.com/), release 2013, were run to create the connectograms for each nucleus.

It is worth to mention that, for an accurate extraction of streamlines of interest, and to avoid erroneous track assignation to a given structure of the basal ganglia, appropriate region of avoidances (*exclude* option of tckedit command) were used (Verstynen et al., 2011).

Connectivity density of the pathways of interest has been estimated by the region-to-region number of streamlines. To summarize the distribution of the connectivity density for each reconstructed pathway we calculated the median density (δ) and standard deviation (*SD*) from individual subject profiles. Analysis of coefficient of variation (COV), defined as the ratio of the *SD* to the δ (COV = *SD*/δ), was also carried out to assess inter-subjects variability.

## Results

By using probabilistic CSD tractography, we reconstructed the undirected connectivity patterns for each nucleus of interest with several supra- and sub-tentorial brain regions. The choice of a proper cutoff for networks is matter of debate in the literature (van Wijk et al., 2010) and probabilistic tractography may yield to spurious results (Rubinov and Sporns, 2010). For these reasons, only connections whose δ was on average above 1% have been considered.

**Figure 1:**
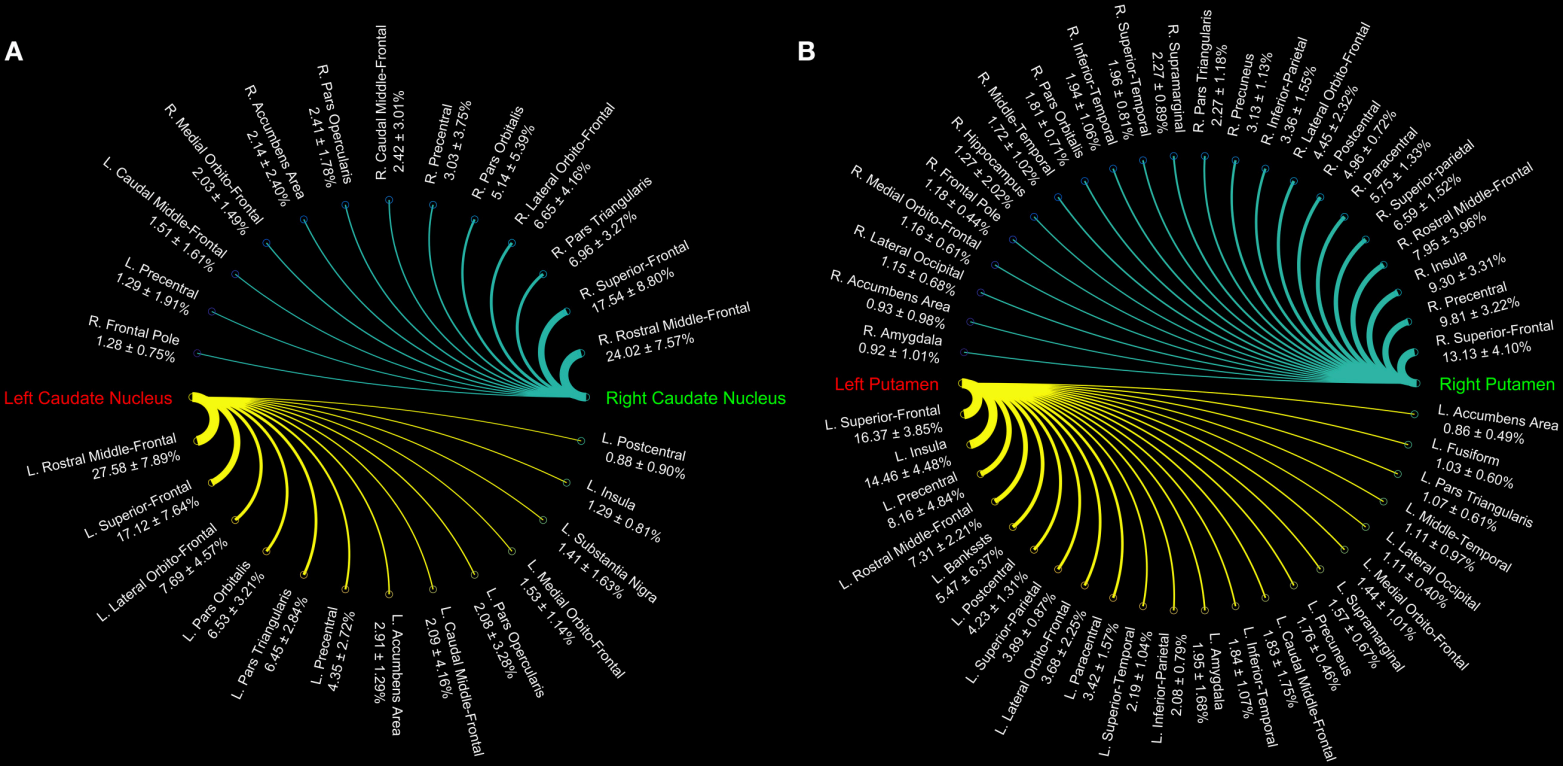
Connectograms of caudate **(A)** and putamen nuclei **(B)**. The circular graphs show the connectivity density profile (median percentages ± standard deviation) for each pathway as provided by tractographic reconstruction in 15 healthy individuals. The line thickness varies according to the connections density.

**Figure 2:**
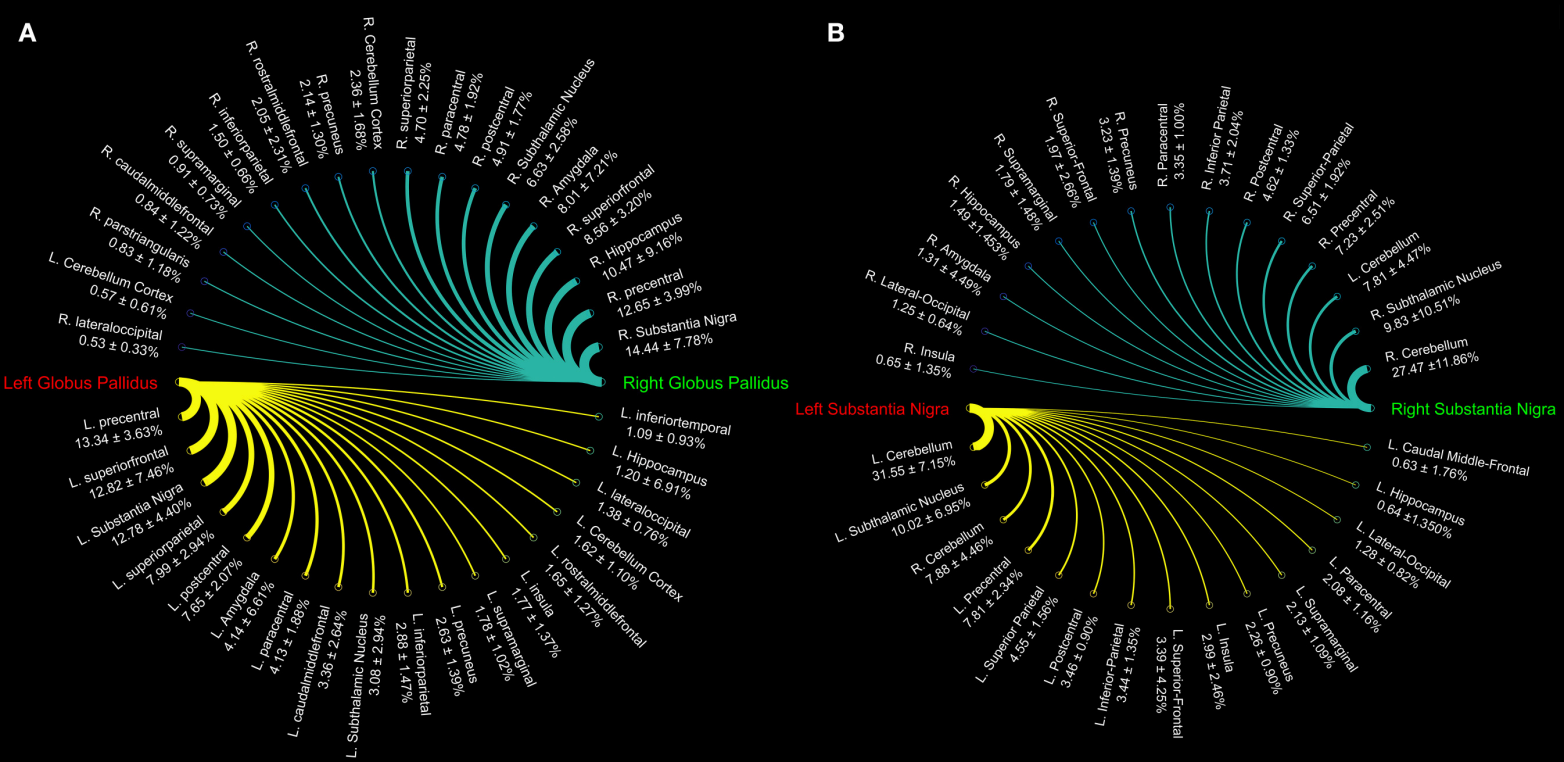
Connectograms showing the connectivity density profile of globus pallidus **(A)** and substantia nigra **(B)** of the right and left sides.

**Figure 3:**
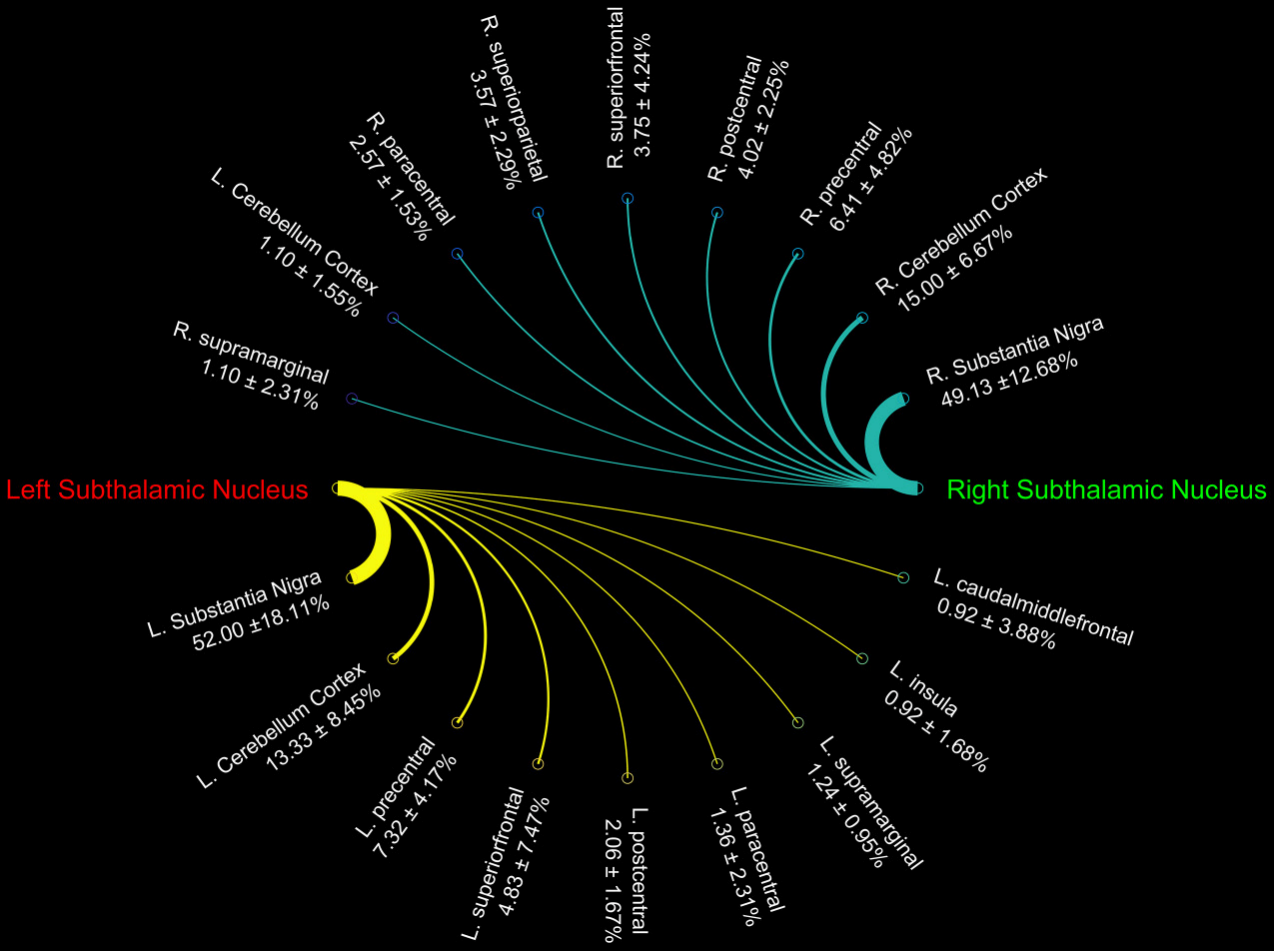
Connectograms showing the connectivity density profile of right and left subthalamic nuclei.

Connectograms estimated for caudate nucleus (Figure 1A), putamen (Figure 1B), GP (Figure 2A), SN (Figure 2B), and STN (Figure 3) are shown for visualization purposes.

In particular, we found that the neostriatum showed an extensive fronto-parietal network including many motor and non-motor regions such as the precentral, postcentral, paracentral, inferior, middle, and superior frontal gyrus as well as the insula, lateral, and medial orbitofrontal cortices (Table 1).

**Table 1:** Summary of connectomic analysis (in percentages) of caudate nucleus and putamen.

**Left**				**Right**			
**Structure**	**Median**	***SD***	**COV**	**Structure**	**Median**	***SD***	**COV**
**CAUDATE NUCLEUS**							
Left rostral middle frontal	27.58	7.89	0.29	Right rostral middle frontal	24.02	7.57	0.32
Left superior frontal	17.12	7.64	0.45	Right superior frontal	17.54	8.80	0.50
Left lateral orbitofrontal	7.69	4.57	0.59	Right pars triangularis	6.95	3.27	0.47
Left pars orbitalis	6.53	3.21	0.49	Right lateral orbitofrontal	6.65	4.16	0.63
Left pars triangularis	6.45	2.84	0.44	Right pars orbitalis	5.14	5.39	1.05
Left precentral	4.35	2.72	0.63	Right precentral	3.03	3.75	1.24
Left accumbens area	2.91	1.29	0.44	Right caudal middle frontal	2.42	3.01	1.24
Left caudal middle frontal	2.09	4.16	1.99	Right pars opercularis	2.41	1.78	0.74
Left pars opercularis	2.08	3.28	1.58	Right accumbens area	2.14	2.40	1.12
Left medial orbitofrontal	1.53	1.14	0.74	Right medial orbitofrontal	2.03	1.49	0.73
Left substantia nigra	1.41	1.63	1.16	Left caudal middle frontal	1.51	1.61	1.06
Left insula	1.29	0.81	0.63	Left precentral	1.29	1.91	1.48
Left postcentral	0.88	0.90	1.03	Right frontal pole	1.28	0.75	0.59
**PUTAMEN**							
Left superior frontal	16.37	3.85	0.24	Right superior frontal	13.13	4.10	0.31
Left insula	14.46	4.48	0.31	Right precentral	9.81	3.22	0.33
Left precentral	8.16	4.84	0.59	Right insula	9.30	3.31	0.36
Left rostral middle frontal	7.31	2.21	0.30	Right rostral middle frontal	7.95	3.96	0.50
Left bankssts	5.47	6.37	1.16	Right superior parietal	6.59	1.52	0.23
Left postcentral	4.23	1.31	0.31	Right paracentral	5.75	1.33	0.23
Left superior parietal	3.89	0.87	0.22	Right postcentral	4.96	0.72	0.14
Left lateral orbitofrontal	3.68	2.25	0.61	Right lateral orbitofrontal	4.45	2.32	0.52
Left paracentral	3.42	1.57	0.46	Right inferior parietal	3.36	1.55	0.46
Left superior temporal	2.19	1.04	0.48	Right precuneus	3.13	1.13	0.36
Left inferior parietal	2.08	0.79	0.38	Right pars triangularis	2.27	1.18	0.52
Left amygdala	1.95	1.68	0.86	Right supramarginal	2.27	0.89	0.39
Left inferior temporal	1.84	1.07	0.58	Right superior temporal	1.96	0.81	0.41
Left caudal middleFrontal	1.83	1.75	0.95	Right inferior temporal	1.94	1.06	0.55
Left precuneus	1.76	0.46	0.26	Right pars orbitalis	1.81	0.71	0.39
Left supra marginal	1.57	0.67	0.43	Right middle temporal	1.72	1.02	0.59
Left medial orbitofrontal	1.44	1.01	0.70	Right hippocampus	1.27	2.02	1.59
Left lateral occipital	1.11	0.40	0.36	Right frontal pole	1.18	0.44	0.37
Left middle temporal	1.11	0.97	0.88	Right medial orbitofrontal	1.16	0.61	0.53
Left pars triangularis	1.07	0.61	0.57	Right lateral occipital	1.15	0.68	0.59
Left fusiform	1.03	0.60	0.58	Right accumbens area	0.93	0.98	1.05
Left accumbens area	0.86	0.49	0.57	Right amygdala	0.92	1.01	1.10

*Standard deviation (SD) and coefficient of variation (COV) are reported for each structure*.

Topographically mapping of GP revealed connectivity patterns with sensory-motor regions, the cerebellum and temporal structures such as the hippocampus and the amygdala. In addition, the nigro-pallidal and pallidal-subthalamic pathways emerged from the connectivity analysis (Table 2).

**Table 2:** Summary of connectomic analysis (in percentages) of globus pallidus.

**Left**				**Right**			
**Structure**	**Median**	***SD***	**COV**	**Structure**	**Median**	**SD**	**COV**
**GLOBUS PALLIDUS**							
Left precentral	13.34	3.63	0.27	Right substantia nigra	14.44	7.78	0.54
Left superior frontal	12.82	7.46	0.58	Right precentral	12.65	3.99	0.32
Left substantia nigra	12.78	4.40	0.34	Right hippocampus	10.47	9.16	0.88
Left superior parietal	7.99	2.94	0.37	Right superior frontal	8.56	3.20	0.37
Left postcentral	7.65	2.07	0.27	Right amygdala	8.01	7.21	0.90
Left amygdala	4.14	6.61	1.60	Right subthalamic nucleus	6.63	2.58	0.39
Left paracentral	4.13	1.88	0.46	Right postcentral	4.91	1.77	0.36
Left caudal middle frontal	3.36	2.64	0.78	Right paracentral	4.78	1.92	0.40
Left subthalamic nucleus	3.08	2.94	0.95	Right superior parietal	4.69	2.25	0.48
Left inferior parietal	2.88	1.47	0.51	Right cerebellum cortex	2.36	1.68	0.71
Left precuneus	2.63	1.39	0.53	Right precuneus	2.14	1.30	0.61
Left supramarginal	1.78	1.02	0.58	Right rostral middle frontal	2.05	2.31	1.13
Left insula	1.77	1.37	0.78	Right inferior parietal	1.50	0.66	0.44
Left rostral middle frontal	1.65	1.27	0.77	Right supramarginal	0.91	0.73	0.80
Left cerebellum cortex	1.62	1.10	0.68	Right caudal middle frontal	0.84	1.22	1.44
Left lateral occipital	1.38	0.76	0.55	Right pars triangularis	0.83	1.18	1.42
Left hippocampus	1.20	6.91	5.75	Left cerebellum cortex	0.57	0.61	1.08
Left inferior temporal	1.09	0.93	0.85	Right lateral occipital	0.53	0.33	0.63

*Standard deviation (SD) and coefficient of variation (COV) are reported for each structure*.

We also evaluated the connectivity profile of the SN which showed the strongest connectivity pattern with both ipsi- and contralateral cerebellum, thus confirming the presence of several parallel cerebellar-basal ganglia circuits and reinforcing the hypothesis of a cerebello-nigral pathway in humans (Milardi et al., 2016a). In addition, SN connectivity was directed toward several fronto-temporo-parietal areas such as the precentral, postcentral, paracentral, superior, inferior parietal gyri, hippocampus, and amygdala (Table 3).

**Table 3:** Summary of connectomic analysis (in percentages) of substantia nigra and subthalamic nucleus.

**Left**				**Right**			
**Structure**	**Median**	***SD***	**COV**	**Structure**	**Median**	***SD***	**COV**
**SUBSTANTIA NIGRA**							
Left cerebellum cortex	31.55	7.15	0.23	Right cerebellum cortex	27.47	11.86	0.43
Left subthalamic nucleus	10.02	6.95	0.69	Right subthalamic nucleus	9.83	10.51	1.07
Right cerebellum cortex	7.87	4.46	0.57	Left cerebellum cortex	7.81	4.47	0.57
Left precentral	7.81	2.34	0.30	Right precentral	7.23	2.51	0.35
Left superior parietal	4.55	1.56	0.34	Right superior parietal	6.51	1.92	0.29
Left postcentral	3.46	0.90	0.26	Right postcentral	4.62	1.33	0.29
Left inferior parietal	3.44	1.35	0.39	Right inferior parietal	3.71	2.04	0.55
Left superior frontal	3.39	4.25	1.25	Right paracentral	3.35	1.00	0.30
Left insula	2.99	2.46	0.82	Right precuneus	3.23	1.39	0.43
Left precuneus	2.26	0.90	0.40	Right superior frontal	1.97	2.66	1.35
Left supramarginal	2.13	1.09	0.51	Right supramarginal	1.79	1.48	0.83
Left paracentral	2.08	1.16	0.56	Right hippocampus	1.49	1.45	0.97
Left lateral occipital	1.28	0.82	0.64	Right amygdala	1.31	4.49	3.44
Left hippocampus	0.64	1.35	2.11	Right lateral occipital	1.25	0.64	0.51
Left caudal middle frontal	0.63	1.76	2.81	Right insula	0.65	1.35	2.08
**SUBTHALAMIC NUCLEUS**							
Left substantia nigra	52.00	18.11	0.35	Right substantia nigra	49.13	12.68	0.26
Left cerebellum cortex	13.33	8.45	0.63	Right cerebellum cortex	15.00	6.67	0.44
Left precentral	7.32	4.17	0.57	Right precentral	6.41	4.82	0.75
Left superior frontal	4.83	7.47	1.55	Right postcentral	4.02	2.25	0.56
Left postcentral	2.06	1.67	0.81	Right superior frontal	3.75	4.24	1.13
Left paracentral	1.36	2.31	1.70	Right superior parietal	3.57	2.29	0.64
Left supramarginal	1.24	0.95	0.76	Right paracentral	2.57	1.53	0.60
Left insula	0.92	1.68	1.83	Left cerebellum cortex	1.10	1.55	1.41
Left caudal middle frontal	0.92	3.88	4.24	Right supramarginal	1.10	2.31	2.11

*Standard deviation (SD) and coefficient of variation (COV) are reported for each structure*.

Finally, STN showed the strongest connections with the SN, probably due to their adjacent anatomical location, as well as many connectivity patterns with fronto-parietal regions involved in the hyperdirect cortico-subthalamic pathway, such as the precentral, postcentral, superior frontal gyri. In addition, in line with previous findings in animals and humans, STN connectivity analysis revealed a strong interaction with the cerebellar cortex (Table 3).

We investigated the consistency of density percentages estimated from our subjects by looking at the COV. While the individual results are reported in Tables 1–3, summary gathered from all structures are shown for a better overview in Table 4. It emerged that the most consistent results were obtained for the putamen, both for the left and right based connectomes (Table 4). Higher variability for the left GP results was observed with respect to the right one, with an average COV of 0.92 vs. 0.72 respectively. An inverse pattern was instead observed for SN, with a slight increment of the variability of the right based connectomes over the left ones (0.90 vs. 0.79 on average). The highest variability between subjects was instead observed for STN connectomes, especially in the left side (Table 4).

**Table 4:** Coefficient of variation (COV) analysis of connectomes estimated from our subjects.

**Structure**	**Side**	**COVs**	
		**Mean**	***SD***
Caudate nucleus	Left	0.80	0.50
	Right	0.86	0.36
Putamen nucleus	Left	0.54	0.25
	Right	0.52	0.33
Globus pallidus	Left	0.92	1.24
	Right	0.72	0.36
Substantia nigra	Left	0.79	0.74
	Right	0.90	0.86
Subthalamic nucleus	Left	1.38	1.20
	Right	0.88	0.58

## Discussion

In the present study we investigated the topography and organization of structural connections of the basal ganglia in humans, showing that the cortico-basal ganglia network consists of several, parallel, and segregated pathways. We have also provided further tractographic evidences of the presence of an extensive anatomical network connecting the cerebellum with the basal ganglia, in line with previous studies in animals and humans.

It should be noted that, while tractography is compelling in being applicable *in vivo* non-invasively in humans, in practice it suffers from well-documented technical limitations. First of all, this technique cannot disentangle monosynaptic from polysynaptic connections, being unable to reveal the existence of synapses or gap junctions, in addition to the inability to determine the directionality (afferent-efferent) of fiber tracts (Chung et al., 2011; Parker et al., 2013). Moreover, it is worthy to note that tractography cannot prove that the reconstructed pathways are anatomically accurate, since it is not able to distinguish discrete axonal pathways, whose diameter is typically less than 10 μm. Hence, tractographic findings should be carefully considered (Jbabdi and Johansen-Berg, 2011).

From the technical point of view, the presence of intravoxel geometric heterogeneity, which is typical of more than 90% of white matter voxels (Jeurissen et al., 2013), has been faced by using CSD diffusion model, which is known to better elucidate fiber pathways than DTI (Tournier et al., 2007).

Although our results are plausible if compared with previous anatomical descriptions of these circuits, tractographic results should be interpreted with caution. Especially when making use of probabilistic algorithms for streamline generation, false positives may be reconstructed. In this regard, to make our tractographic findings more consistent, we decided to increase the cutoff for tracking generation and termination, therefore using a conservative approach (Descoteaux et al., 2009; Milardi et al., 2016a,b; Cacciola et al., 2017a). This was accomplished at the cost of an underestimation issue.

In addition, the tracking of parallel segregated pathways throughout the cortico-subcortical circuits may be limited by the overlapping nature of the basal ganglia. We tried to limit such issue by combination of inclusion ROIs and regions of avoidance (ROAs).

On the other hand it is worthy to note that it has been demonstrated that this technique permits to obtain good macroscopic neuroanatomical information on white matter fiber bundles by reconstructing streamlines structures containing bundles of axons running along the same direction (Mori and Van Zijl, 2002).

In conclusion, tractography is an anatomical technique by which functional significance can be hypothesized. However, it may provide an interesting perspective for studying altered connectivity patterns in neuropsychiatric and movement disorders related to the cortico-basal ganglia-cerebellar connectome.

### Cortico-striatal connectivity

Cortico-striatal pathway has been traditionally described as a rich set of connections between the putamen as well as the caudate head and body with frontal, parietal and, rarely, occipital regions. Our results are substantially in line with previous findings showing that caudate head and body are principally connected with frontal areas such as the ventral prefrontal cortex, superior frontal gyrus (SFG), and rostral middle frontal gyrus (rMFG) (Lehéricy et al., 2004b). In addition, we here showed orbitofrontal-striatal connectivity, but, in agreement with previous findings (Draganski et al., 2008), no suprathreshold connections were found for the paracentral lobule and premotor area with caudate head and body.

On the other hand, the present findings of a strong connectivity between the precentral gyrus and putamen are in line with previous data showing that connections with motor areas preferentially involve the putamen rather than the caudate nucleus (Lehéricy et al., 2004a).

These results suggest that the head and body of caudate nucleus are mainly involved in the so-called “associative-cognitive loop,” being preferentially connected with areas involved in self-awareness (SFG) (Goldberg et al., 2006), in cognitive processes (dlPFC) (Cieslik et al., 2013), in memory retrieval and language (pars opercularis, pars orbitalis, and pars triangularis) (Kostopoulos and Petrides, 2003; Friederici, 2011).

In addition, a functional striatal connectivity with several frontal areas has been demonstrated, suggesting that the dorsal caudate nucleus is more closely related to the lateral orbitofrontal cortex (OFC) than to medial portion (Di Martino et al., 2008). In line with these findings, the structural connectivity analysis of the present study indeed revealed stronger caudate connectivity patterns with the lateral OFC than with the medial one.

On the other hand, we here confirmed that the putamen receives its major input from parietal areas linked with sensorimotor functions. In addition, putamen is connected with some different associative regions such as the superior temporal gyrus (STG), supramarginal and lingual gyri, and insula. In particular, STG connections with putamen may be considered substantially as the human analog of those described in *Cebus Apella* monkeys using HSV strains as retrograde fiber tracer (Middleton and Strick, 1996). Connections between putamen and pericalcarine and lingual gyri and between putamen and left superior parietal lobule have been described in human brain in a combined resting state-functional MRI and DTI study, showing that FA value changes in these pathways correlated with cognitive processing speed in healthy humans (Ystad et al., 2011).

Although similar considerations can be made for the functional connectivity of putamen with sensorimotor cortices and with insula, we were not able to match the significant functional connectivity reported for anterior cingulate cortex with both caudate and putamen (δ < 1%) (Di Martino et al., 2008).

### Cortico-STN connectivity

Although the STN is considered one of the relevant nodes of the “indirect” pathway, it also receives a direct input from the cerebral cortex (Kitai and Deniau, 1981; Nambu et al., 2000). Indeed, Nambu et al. (2002) described a glutamatergic direct cortical input on the STN, conveying excitatory stimuli from motor, associative and limbic brain areas on the GP, bypassing the indirect inhibitor circuit. In line with these findings, by using 7T MRI and tractography, it has been demonstrated that the posterior medial frontal cortex-STN white matter tract strength predicts inter-individual efficacy in stopping a movement in a motor no-go task (Forstmann et al., 2012). Herein, we showed that the connectivity density of the STN is mainly distributed to the precentral, postcentral gyri, and the paracentral lobule, which are cortical areas involved in sensorimotor functions. Moreover, the connectivity pattern linking STN and SFG detected in the present study is in line with the recent idea of a “cognitive STN” involved in decision-making (Weintraub and Zaghloul, 2013).

### Cortico-pallidal connectivity

Although the cortical inputs are traditionally thought to reach the GP via the direct, indirect, and hyperdirect pathways, there is growing evidence, in animal studies, of the possible existence of a direct route connecting the GP and the cerebral cortex reciprocally. In an anterograde tract tracing study, Naito and Kita (1994) described the presence of a cortico-pallidal projection (which accounted for about 10% of the corticostriatal pathway density) in rodents, linking the medial and lateral precentral cortices to the GPe. More recently, it has been reported that in turn cholinergic and GABAergic neurons within the GPe send direct efferent to the cortex (Chen et al., 2015; Saunders et al., 2015). This suggests a possible major revision of basal ganglia circuits in which GP projections toward the frontal cortex may modulate the activity of different cortical areas. Furthermore, Milardi et al. (2015) highlighted the possible existence of a direct cortico-pallidal pathway in humans based on CSD-tractography; its function, however, remains to be clarified.

In the present study, we provide, for the first time, a quantitative characterization of the GP structural connectivity with the cerebral cortex. Our connectivity analysis showed an anatomical pallidal-temporal network involving the hippocampus and amygdala, as well as a sensorimotor-pallidal network involving mainly the precentral, postcentral, and paracentral gyrus, in addition to high-order functions-related areas such as the SFG.

Taking into account that our approach used the caudate, putamen, and thalamic nuclei as exclusion masks in order to avoid the reconstruction of the short subcortical connections within the subcortical basal ganglia network, the described connectivity patterns might reflect the direct information flow between the cerebral cortex and GP, in line with previous animal and human studies (Naito and Kita, 1994; Chen et al., 2015; Milardi et al., 2015; Saunders et al., 2015).

In addition, considering the combination of ROIs and ROAs employed for tract reconstruction, it is likely that the cortico-pallidal pathway constitutes an additional system separate from the cortico-spinal tract and cortico-pontine pathway. Indeed, Smith and Wichmann (2015) in an editorial note suggested the cortico-pallidal route as an additional regulatory glutamatergic pathway in basal ganglia circuitry in rodents, monkeys, and humans.

### Cortico-nigral connectivity

Only a few studies have investigated so far the structural connectivity of SN in the human brain using DTI. Two nigral regions have been identified on the basis of connectivity patterns (Menke et al., 2010): an internal region (likely corresponding to the SNc) which was mainly connected with the striatum, GP, anterior thalamus, and prefrontal cortex, as well as an external region showing major connectivity with posterior thalamus, ventral thalamus, and motor cortex, which likely corresponded to the SNr, in agreement with the anatomical knowledge (Parent and Hazrati, 1994). More recently Kwon and Jang (2014) evaluated differences in structural connectivity between SN and ventral tegmental area, showing that SN has an extensive network with many frontal, parietal, temporal areas as well as with the cerebellum. We have recently hypothesized the possible existence of a direct cortico-nigral pathway connecting SN with many cortical areas related to motor functions, such as precentral gyrus, postcentral gyrus, and paracentral lobule as well as to the SFG in the prefrontal cortex (Cacciola et al., 2016a).

In the present study the whole brain-based connectivity analysis revealed that the SN is mainly connected with the precentral, postcentral gyri, and paracentral lobules, in addition to frontal and parietal areas involved both in sensorimotor and high order functions. If, on the one hand, the connectivity patterns between prefrontal cortex and SN are consistent with previous findings in primates (Frankle et al., 2006), the sparse connectivity described between OFC and SN in primates was not found to be sufficiently dense in the present study, in line with our previous results (Cacciola et al., 2016a). The presence of an intricate network between the cerebral cortex and SN might play a very important role in the basal ganglia circuitry, as it may play as an upstream control on basal ganglia response by modulating the nigro-striatal system.

It is worthy to note that beyond the strong connectivity between SN and STN, which is likely to be heavily influenced by their close anatomical location, SN revealed consistent connections with the cerebellum, which are the subject of the next section.

### Cerebellum-basal ganglia connectivity

Last but not least, we here highlighted extensive parallel subcortical loops between the basal ganglia and the cerebellum. Several studies have suggested that cerebellum and basal ganglia are strongly interconnected in physiological and pathological conditions.

In line with previous studies (Bostan et al., 2010, 2013), we here provided further support to the existence of a pathway running between the STN and cerebellar cortex. Indeed, we found that STN showed a strong connectivity profile with the ipsilateral cerebellar cortex. The weaker connectivity observed for the contralateral connections might be due the longer distance covered by these pathways to reach their final targets. Although from tractographic reconstruction we cannot infer about directionality, this pathway may correspond to STN efferents toward the cerebellar cortex demonstrated in monkeys using retrograde virus-tracing technique (Bostan et al., 2010).

In the present study, the whole brain-based connectivity analysis revealed that, at the level of the basal ganglia, the cerebellum interacts not only with the STN but with the GP as well.

Our results show dense connectivity between SN and both ipsilateral and contralateral cerebellar cortex. Our findings are in line with previous fMRI study which demonstrated a bilateral connectivity between SNc and cerebellar vermis (Tomasi and Volkow, 2014) as well as posterior and lateral cerebellar cortex (Zhang et al., 2016). Indirect evidence for a structural-functional interaction between SN and cerebellum comes also from studies in animals which suggested that the focal electrical stimulation of the dentate and fastigial nuclei leads to fluctuation in dopamine turnover in the SN (Nieoullon et al., 1978).

## Concluding remarks

The last decade has been characterized by the growing idea that, in addition to the direct, indirect, and hyperdirect pathways, several other circuits can contribute to modulate the basal ganglia functioning. Herein, we performed a connectivity analysis on diffusion MRI data in order to characterize the topographical distribution of the connections involved in the cortico-basal ganglia-cerebellar network.

Besides confirming the dense striatal and subthalamic structural connectivity with several frontal and parietal areas, we here showed that the GP and SN are densely interconnected with the cerebral cortex as well. Although we cannot provide any direct evidence on the functional significance of these connections, the findings lead to speculate that the sensorimotor-pallidal pathway plays a relevant role in motor control within the cortico-basal ganglia-cortical loop, allowing for a faster information flow between the cerebral cortex and the basal ganglia with respect to the direct, indirect and hyperdirect pathways, as previously hypothesized (Cacciola et al., 2016b).

In addition, our analysis revealed that the STN, GP, and SN are structurally interconnected with the cerebellum. These findings reinforce the idea that the basal ganglia are part of an extensive intricate network with the cerebellum thus mediating the afferent and efferent information flow to integrate inputs of different modalities and to rapidly provide an adaptive motor response (Cacciola et al., 2017b).

Finally, it is worth to note that the interplay between the basal ganglia and cerebellum may be relevant in several movement disorders such as dystonia and Parkinson's disease considering the emergent idea of a compensatory and modulatory function of the cerebellum either at the early stages of the disease or during its progression (Wu and Hallett, 2013; Neumann et al., 2015; Chillemi et al., 2017).

Our findings may pave the way for a more comprehensive and holistic pathophysiological model of basal ganglia circuits.

## Author contributions

AlbC: Study concepts/study design, data analysis, data interpretation, literature research, and draft the manuscript. AleC: Study concepts/study design, data analysis, and draft the manuscript. DM: Data acquisition, data interpretation, and literature research. EM, GC, and AN: Data analysis, data interpretation, and literature research. SM: Data acquisition and data interpretation. GR: Data interpretation and literature research. GA: Guarantor of integrity of entire study, data interpretation, and manuscript revision for important intellectual content. AQ: Study concepts/study design, guarantor of integrity of entire study, and manuscript revision for important intellectual content. All the authors approved the final version of the manuscript.

### Conflict of interest statement

The authors declare that the research was conducted in the absence of any commercial or financial relationships that could be construed as a potential conflict of interest.
